# Divergence in the Regulation of the Salt Tolerant Response Between *Arabidopsis thaliana* and Its Halophytic Relative *Eutrema salsugineum* by mRNA Alternative Polyadenylation

**DOI:** 10.3389/fpls.2022.866054

**Published:** 2022-03-25

**Authors:** Hui Ma, Lingling Cai, Juncheng Lin, Kaiyue Zhou, Qingshun Q. Li

**Affiliations:** ^1^Key Laboratory of the Ministry of Education for Coastal and Wetland Ecosystems, College of the Environment and Ecology, Xiamen University, Xiamen, China; ^2^Graduate College of Biomedical Sciences, Western University of Health Sciences, Pomona, CA, United States

**Keywords:** alternative polyadenylation, salt tolerance, *Arabidopsis thaliana*, *Eutrema salsugineum*, PAT-seq, RNA processing

## Abstract

Salt tolerance is an important mechanism by which plants can adapt to a saline environment. To understand the process of salt tolerance, we performed global analyses of mRNA alternative polyadenylation (APA), an important regulatory mechanism during eukaryotic gene expression, in *Arabidopsis thaliana* and its halophytic relative *Eutrema salsugineum* with regard to their responses to salt stress. Analyses showed that while APA occurs commonly in both *Arabidopsis* and *Eutrema*, *Eutrema* possesses fewer APA genes than *Arabidopsis* (47% vs. 54%). However, the proportion of APA genes was significantly increased in *Arabidopsis* under salt stress but not in *Eutrema*. This indicated that *Arabidopsis* is more sensitive to salt stress and that *Eutrema* exhibits an innate response to such conditions. Both species utilized distal poly(A) sites under salt stress; however, only eight genes were found to overlap when their 3′ untranslated region (UTR) lengthen genes were compared, thus revealing their distinct responses to salt stress. In *Arabidopsis*, genes that use distal poly(A) sites were enriched in response to salt stress. However, in *Eutrema*, the use of poly(A) sites was less affected and fewer genes were enriched. The transcripts with upregulated poly(A) sites in *Arabidopsis* showed enriched pathways in plant hormone signal transduction, starch and sucrose metabolism, and fatty acid elongation; in *Eutrema*, biosynthetic pathways (stilbenoid, diarylheptanoid, and gingerol) and metabolic pathways (arginine and proline) showed enrichment. APA was associated with 42% and 29% of the differentially expressed genes (DE genes) in *Arabidopsis* and *Eutrema* experiencing salt stress, respectively. Salt specific poly(A) sites and salt-inducible APA events were identified in both species; notably, some salt tolerance-related genes and transcription factor genes exhibited differential APA patterns, such as *CIPK21* and *LEA4-5*. Our results suggest that adapted species exhibit more orderly response at the RNA maturation step under salt stress, while more salt-specific poly(A) sites were activated in *Arabidopsis* to cope with salinity conditions. Collectively, our findings not only highlight the importance of APA in the regulation of gene expression in response to salt stress, but also provide a new perspective on how salt-sensitive and salt-tolerant species perform differently under stress conditions through transcriptome diversity.

## Introduction

Salt stress is a major global issue for agricultural production. More than 800 million hectares of cultivated land is affected by high salinity (Munns and Tester, 2008). Rising salt concentration in soil or water can have a significant detrimental effect on crop yields. Excess salt represents a major threat to germination, growth, and the production of plants in saline soil. Understanding how plants respond to salt conditions and the molecular mechanisms of salt tolerance is important for stress biology research and also meaningful for genetic improvements of salt resistance in crops.

*Eutrema salsugineum* is closely related to *Arabidopsis thaliana* but it can grow in natural harsh environments. *Eutrema* is widely used as a model system to investigate how plants cope with high salinity, extreme cold, and water shortage (Khanal et al., 2015; Li et al., 2021a). Although the divergence time between *Eutrema* and *Arabidopsis* is approximately 43.2 MYA, these plants share over 80% of genes and exhibit highly homologous orthologs (Yang et al., 2013). How they respond to salt stress differently has been intriguing, and the underlying mechanisms that control salt acclimation at transcriptional level are not well understood.

Messenger RNA polyadenylation is a pre-mRNA processing event that affects gene expression. It involves two main steps: cleavage of the 3′ end of pre-mRNAs by polyadenylation factors and the addition of a poly(A) tail, which bridges other transcriptional and post-transcriptional processes, such as splicing (Deng and Cao, 2017), and transcriptional termination (Antosz et al., 2017). It has been reported that plant genes possess multiple polyadenylation sites, and over 70% of genes in *Arabidopsis* and rice are alternatively polyadenylated (Wu et al., 2011; Berkovits and Mayr, 2015; Fu et al., 2016; Kim et al., 2016). Alternative polyadenylation (APA) can enhance the diversity of the transcriptome, affect mRNA stability, export, localization, and influence translation processes (Xing and Li, 2011). Genome-wide APA dynamics in development and stress responses have been reported in several species of plants, including *A. thaliana* (Yu et al., 2019), *Oryza sativa* (Fu et al., 2016), *Medicago truncatula* (Wu et al., 2014), *Sorghum bicolor* (Abdel-Ghany et al., 2016), bamboo (Wang et al., 2017), and algae like *Chlamydomonas reinhardtii* (Zhao et al., 2014) and diatom (Fu et al., 2019).

Alternative polyadenylation is tightly associated with many environmental responses in plants, including oxidative stress (Zhang et al., 2008), hypoxia (de Lorenzo et al., 2017), drought (Ye et al., 2019), heat (Chakrabarti et al., 2020), and heavy metal stresses (Cao et al., 2019). Several studies on polyadenylation factors, including CPSF30, FIP1, and FY, suggested that polyadenylation factors-mediated APA is important for stress responses (Chakrabarti and Hunt, 2015; Tellez-Robledo et al., 2019; Yu et al., 2019). Previous research has indicated that APA is involved in the expression of genes related to salt tolerance. For example, *AtSOT12* exhibits salt-inducible expression and the manner in which the poly(A) site is used has been shown to change under conditions of salt stress, thus identifying novel mechanisms of salt-responsive gene regulation (Chen et al., 2015). It was demonstrated that transcripts of *AtARK2* and a zinc ion binding protein generated by APA play roles in salt and oxidative stress responses (Yu et al., 2019). Besides, *Sorghum* showed APA-mediated transcriptome remodeling in response to salt stress (Chakrabarti et al., 2020).

Here, we performed high-throughput poly(A) tag sequencing (PAT-seq) with a salt-sensitive species *A. thaliana* and a salt-tolerant species *E. salsugineum* when treated with 200 mM of NaCl. We provided a comprehensive map of poly(A) profiles of the two species under salt conditions, identified differential gene expression patterns and distinct poly(A) profiles, and revealed a new perspective on the potential role of APA in plant response to salt stress.

## Materials and Methods

### Plant Materials and Salt Stress Treatments

*Arabidopsis thaliana* (ecotype: Col-0; CS60000) and *Eutrema salsugineum* (ecotype: Shandong; formerly known as *Thellungiella halophila*; thus, the gene names were still in prefix *Thhalv* according to its genome annotation files) were used for root growth phenotyping. Seeds were sterilized with sodium hypochlorite for 3 min and rinsed with distilled water for five times. Then, seeds were synchronized at 4°C in the dark for 3 days (*Arabidopsis*) or for 7 days (*Eutrema*). *Eutrema* seeds were sterilized 4 days ahead of *Arabidopsis* seeds so that they could be sowed at the same time. Seeds were sowed on 1/2 Murashige and Skoog (MS) medium (with 2% sucrose) and placed vertically in a growth chamber with 16 h-light and 8 h-dark cycles at 21 ± 1°C for seedling growth. Five-day-old seedlings were transferred onto 1/2 MS medium containing 0, 50, 150, 200, or 300 mM NaCl, and the positions of the root tips were marked. Photographs were taken 8 days later and tap root elongation was determined by Image J. Three biological replicates were performed for each concentration, and each replicate contained five seedlings.

For short-term treatment, *Arabidopsis* and *Eutrema* seeds were sterilized and synchronized as described above and then sowed on 1/2 MS medium and kept for 13 days to allow vertical growth. Then, the seedlings were transferred to 1/2 MS medium containing 0 or 200 mM NaCl and treated for 3 h. Next, whole seedlings were immediately frozen in liquid nitrogen and stored at −80°C until RNA extraction. Three biological replicates were performed, and six seedlings were pooled into each replicate.

### PAT-seq Library Preparation

Total RNA was extracted with a TaKaRa MiniBEST Plant RNA Extraction Kit and genomic DNA was removed by DNaseI (New England Biolabs). PAT-seq libraries were prepared as previously described with modifications (Lin et al., 2020). Two micrograms of total RNA were fragmented by 5× first strand buffer (TaKaRa) at 94°C for 4 min. Poly(A) RNAs were then enriched by oligo(dT)_25_ beads (New England Biolabs). Reverse transcription was performed with oligo d(T)_18_ primers by SMARTScribe™ Reverse Transcriptase (TaKaRa) for 2 h at 42°C. Then, a modified 5′ adaptor and SMARTScribe Reverse Transcriptase were added for another 2 h at 42°C. The cDNA generated was then purified with AMPure beads and amplified with Phire II (Thermo Fisher Scientific). The amplification products were then separated on a 2% agarose gel and 300–500 bp fragments were purified with a Zymoclean Gel DNA Recovery Kit. The concentration and quality of libraries were tested by a Qubit 2.0 and an Agilent Bioanalyzer 2100, and then sequenced on an Illumina HiSeq 2500 platform with 100-bp rapid sequencing mode.

### Identification of Poly(A) Sites

Raw reads were filtered by the FASTX-Toolkit with a threshold of *q* = 10 and low-quality reads were discarded. The remaining reads were mapped to the *A. thaliana* TAIR10 genome and the *E. salsugineum* genome (Yang et al., 2013) by Bowtie 2 (Langmead and Salzberg, 2012). Poly(A) site analysis was performed as previously described (Lin et al., 2020). Internal priming events were filtered out by custom perl script and poly(A) tags (PATs) within 24 nucleotides (nt) were clustered into one poly(A) cluster (PAC), which represented a poly(A) site. As 70% of the *Eutrema* poly(A) sites were located within 200 nt downstream of the annotated genes (Supplementary Figure S1), we extended the 3′ untranslated region (UTR) by 200 nt to recover the PACs that fell within this region (Wu et al., 2014). In the case of genes that did not have a 3′ UTR annotation, we extended by an extra 218 nt (the average length of 3′ UTRs in *Eutrema*). PACs with less than 10 PATs were filtered out and DEseq2 (Love et al., 2014) was used to normalize PAT counts and analyze differential expression among the samples, an adjusted value of *p* < 0.05 was set as the threshold for significance. PAT-seq coverage of genes was visualized by Integrative Genomics Viewer (IGV) v2.8.3 (Robinson et al., 2011).

### 3′ UTR Length Analysis

The weighted length of the 3′ UTRs in genes was analyzed as described previously (Lin et al., 2020). Genes with at least two PACs in their 3′ UTRs were used to identify shortening and lengthening events in the 3′ UTR. Pearson’s correlation coefficient was used to indicate the strength of 3′ UTR shortening (<0) or 3′ UTR lengthening (>0). Adjusted *p* values from Chi-square tests were used to indicate the significance of changes in the length of the 3′ UTR.

### Gene Ontology and Kyoto Encyclopedia of Genes and Genomes Analysis

Gene ontology (GO) enrichment analysis was performed with agriGO (Tian et al., 2017; http://systemsbiology.cau.edu.cn/agriGOv2/). GO annotation of *Eutrema* was downloaded from http://plantregmap.gao-lab.org/index-chinese.php (Jin et al., 2017). Kyoto Encyclopedia of Genes and Genomes (KEGG) pathway enrichment was performed using KOBAS (Xie et al., 2011; http://kobas.cbi.pku.edu.cn/kobas3/). Gene IDs were converted to Entrez IDs by the Gene ID conversion tool in DAVID (Huang da et al., 2009; https://david.ncifcrf.gov/). An adjusted value of *p* < 0.05 was set as the threshold for significance.

### RT-qPCR Analysis

Two micrograms of DNA-free total RNA were used for reverse transcription. RT-qPCR was performed on a CFX96™ Real-Time PCR Detection System (Bio-Rad) with SYBR green PCR master mix. Primers are shown in Supplementary Table S3. *AtACTIN2* was used as the reference gene for *Arabidopsis* while *EsTUB6* was used as the reference gene for *Eutrema*.

### Statistical Analysis

SPSS R.23.0.0 was used for data analysis; one-way ANOVA and the Least Significant Difference test were used to determine statistical significance. Wilcoxon matched-pairs signed rank test was used to test the significance in boxplot. The mean values and SDs were calculated from three biological replicates. Significant differences were indicated as ^*^p < 0.05; ^**^p < 0.01; ^***^p < 0.001; ^****^p < 10e−04.

### Data Availability

The PAT-seq data generated by this study are available in the NCBI BioProject database^1^ under accession number PRJNA782687.

## Results

### The Growth of *Arabidopsis* and *Eutrema* Roots Under Salt Stress

**Figure 1:**
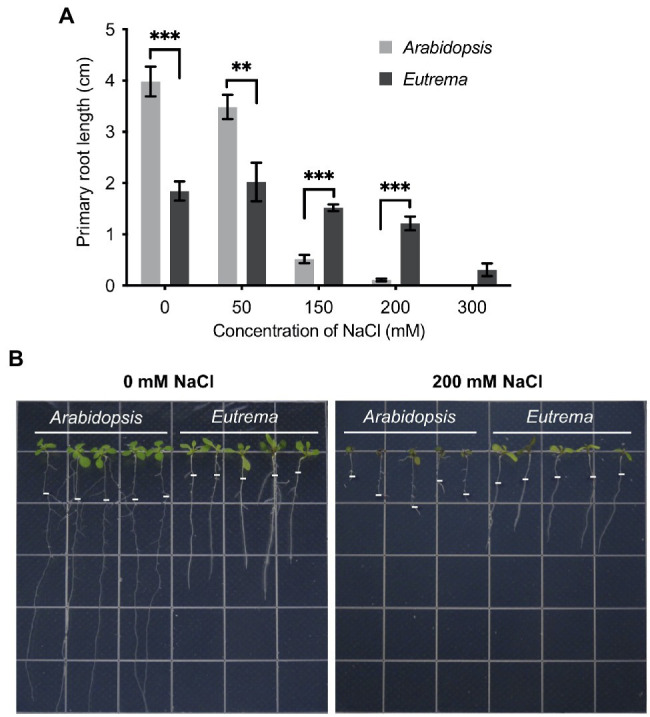
Primary root growth under salt stress in *Arabidopsis* and *Eutrema*. **(A)** Measurements of primary root length after 8 days under salt stress. Statistical significance was determined by one-way ANOVA, ^**^p < 0.01, ^***^p < 0.001. **(B)** Phenotypes of primary root growth under 0 and 200 mM NaCl conditions. The white short lines mark the positions of root tips at Day 0 of the treatment.

Root elongation under salt conditions was measured to evaluate the salt tolerance of *Arabidopsis* and *Eutrema*. Five-day-old seedlings were transferred to 1/2 MS medium containing different concentrations of NaCl (0, 50, 150, 200, and 300 mM) and primary root elongation was measured after 8 days. Under normal condition or a relatively low concentration of NaCl (50 mM), *Arabidopsis* grew longer roots than *Eutrema* (0 mM, *p* < 0.001, 50 mM, *p* < 0.01, Figure 1A). However, under conditions with higher concentrations of NaCl (>150 mM), the root growth of *Arabidopsis* was significantly restricted (*p* < 0.001, Figure 1A). At a NaCl concentration of 200 mM, the elongation of *Arabidopsis* roots was reduced to 2.5% of that at 0 mM NaCl (*p* < 0.001); in comparison, 66% of root growth was maintained in *Eutrema* (Figures 1A,B). These results suggest that *Eutrema* performed significantly better than *Arabidopsis* under salt stress; these findings are consistent with previous studies which showed that *Eutrema* is highly tolerant to salt (Kazachkova et al., 2013). On the basis of these results, we selected 200 mM NaCl for the construction of PAT-seq libraries as most significant differences were seen at this concentration between the two species.

### Profiles of the Poly(A) Sites of *Arabidopsis* and *Eutrema* Under Salt Stress

**Figure 2:**
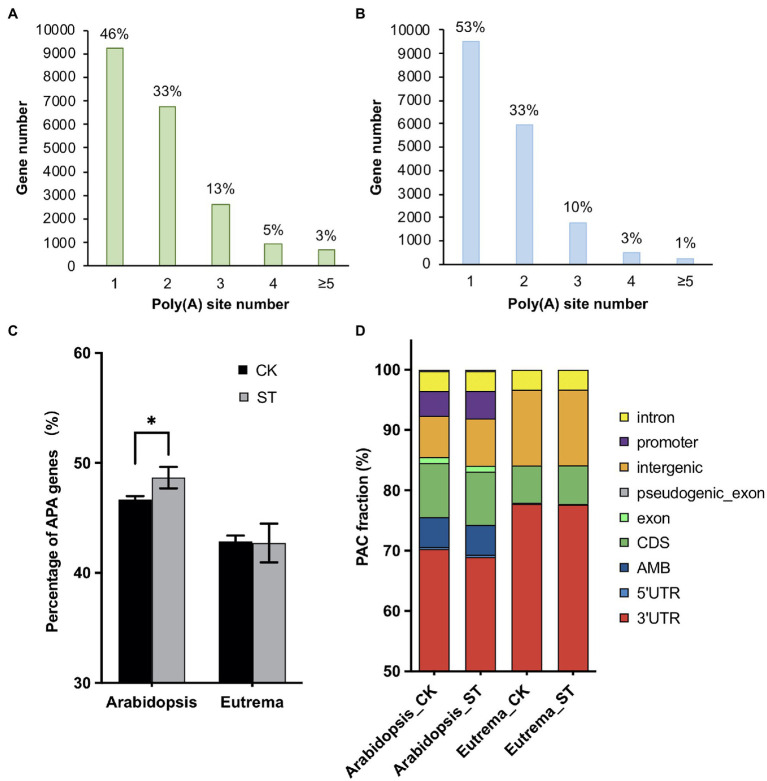
Profiles of the poly(A) sites of *Arabidopsis* and *Eutrema* under control and salt stress conditions. **(A)** Genes and poly(A) sites (per gene) identified in *Arabidopsis*. The *X*-axis shows the number of poly(A) sites per gene. Percentages represent gene fractions. **(B)** Genes and poly(A) sites (per gene) identified in *Eutrema*. **(C)** The ratio of alternatively polyadenylated genes (APA genes) in *Arabidopsis* and *Eutrema* under control (CK) and salt stress (ST) conditions. Statistical significance was determined by one-way ANOVA, ^*^p < 0.05. **(D)** Poly(A) cluster (PAC) distribution among different genomic regions in *Arabidopsis* and *Eutrema* under CK and ST conditions. AMB: ambiguous PACs assigned to more than one genomic region.

To determine poly(A) site profiles (hence APA events) in *Arabidopsis* and *Eutrema* under salt stress, we collected seedlings of the two species under control (CK, 0 mM NaCl) and salt stress (ST, 200 mM NaCl) conditions for PAT-seq. After raw data processing, 44,395 PACs were identified in *Arabidopsis*; these were dispersed amongst 20,208 genes. Of these genes, 54% possessed more than one poly(A) site; these were defined as APA genes (Figure 2A). In contrast, 30,226 PACs were identified in *Eutrema*; these were dispersed in 17,939 genes; 47% of these were classified as APA genes (Figure 2B). These results suggest that APA occurs commonly in *Arabidopsis* and *Eutrema*.

Notably, salt stress induced more than 400 APA genes in *Arabidopsis* while no significant changes were observed in *Eutrema* (Figure 2C), thus indicating that *Arabidopsis* is more sensitive to salt stress. Furthermore, salt stress reduced the proportion of PACs in the 3′ UTRs of *Arabidopsis* but increased those in intergenic regions; however, no such changes were evident in *Eutrema* (Figure 2D), thus suggesting that salt stress induced lower levels of interference in the *Eutrema* transcriptome.

### *Arabidopsis* and *Eutrema* Showed Distinct Poly(A) Profiles and Gene Expression Patterns Under Salt Stress

As *Arabidopsis* and *Eutrema* are known to respond differently to salt stress, we applied principal component analyses to determine specific response patterns. Data reflected the experimental design in that CK samples were clustered together but away from the ST samples in both *Arabidopsis* and *Eutrema* (Supplementary Figure S2), thus indicating that both species exhibit a distinct expression pattern of poly(A) sites under salt stress. Differently expressed PAC (DE-PAC) analysis showed that *Arabidopsis* possessed 3,037 DE-PACs (*p*_adj_ < 0.05; Supplementary Table S1) while *Eutrema* had 998 DE-PACs (*p*_adj_ < 0.05; Supplementary Table S2). These DE-PACs were located in 2,566 and 849 genes, respectively, and were designated as DE-PAC genes.

**Figure 3:**
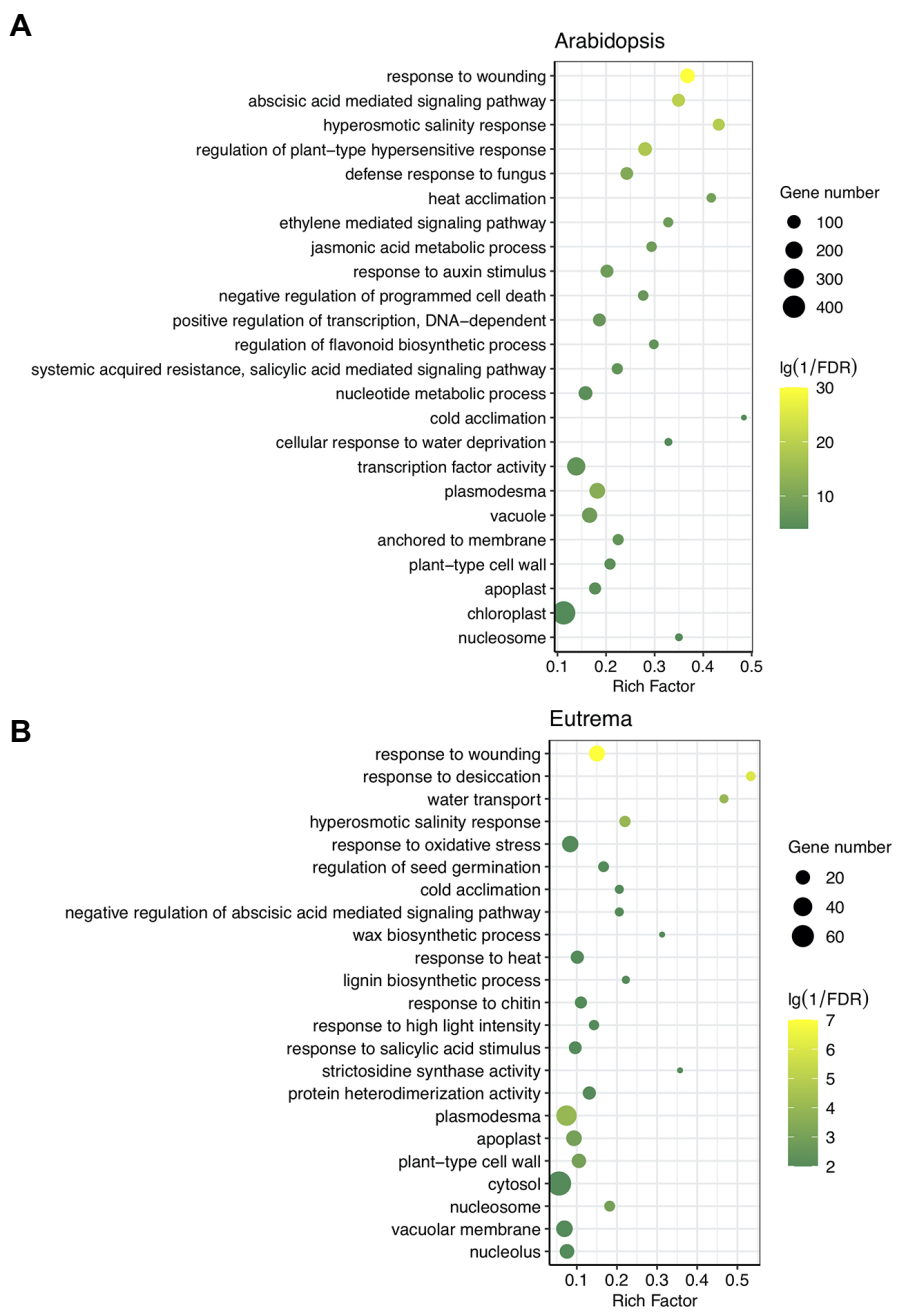
Gene ontology (GO) analysis of differentially expressed PAC genes (DE-PAC genes) in *Arabidopsis* **(A)** and *Eutrema* **(B)**. Rich factor indicates the gene number annotated in the term divide by the gene number in reference annotation. FDR, false discovery rate. The size of the dot indicates the number of genes; the color indicates significance.

Next, we investigated the potential functions of these DE-PAC genes by performing GO enrichment and KEGG pathway analyses. In both species, DE-PAC genes were enriched in a range of biological processes, including hyperosmotic salinity response, hormone-mediated signal pathways, response to wounding, response to heat and cold; and a range of cellular components, including plasmodesma, apoplast, and cell wall (Figure 3). However, several terms of biological processes were identified to be different in the two species, including negative regulation of programmed cell death, positive regulation of transcription, flavonoid biosynthetic process that only showed in *Arabidopsis*; whereas response to oxidative stress, biosynthetic process of wax and lignin only showed in *Eutrema* (Figure 3). Besides, for both *Arabidopsis* and *Eutrema*, DE-PAC genes were enriched in a range of different molecular functions. In *Arabidopsis*, we identified DE-PAC genes that were associated with transcription factors; however, in *Eutrema*, the DE-PAC genes were related to protein heterodimerization activity (Figure 3). Upregulated DE-PAC genes in *Arabidopsis* were significantly enriched in several KEGG pathways, including plant hormone signal transduction, starch and sucrose metabolism, and fatty acid elongation (Table 1). For downregulated DE-PAC genes, no pathways were significantly enriched. However, in *Eutrema*, upregulated DE-PAC genes were significantly enriched in biosynthetic pathways (stilbenoid, diarylheptanoid, and gingerol) and metabolic pathways (arginine and proline). Downregulated DE-PAC genes were enriched in protein processing in the endoplasmic reticulum. Collectively, these results revealed that *Arabidopsis* and *Eutrema* respond to salt stress differently with distinct gene expression profiles; it is likely that they also possess different molecular mechanisms.

**Table 1:** Kyoto Encyclopedia of Genes and Genomes (KEGG) pathway analysis of DE-PAC genes under salt stress in *Arabidopsis* and *Eutrema*.

Pathway ID	Pathway name	Input number	Background number	*Padj*
*Arabidopsis*				
Upregulated				
ath04075	Plant hormone signal transduction	35	271	2.37E−04
ath00500	Starch and sucrose metabolism	26	202	2.32E−03
ath00062	Fatty acid elongation	8	35	2.95E−02
*Eutrema*				
Upregulated				
eus04075	Plant hormone signal transduction	18	291	2.68E−03
eus00945	Stilbenoid, diarylheptanoid, and gingerol biosynthesis	5	36	2.67E−02
eus00330	Arginine and proline metabolism	6	55	2.67E−02
Downregulated				
eus04141	Protein processing in endoplasmic reticulum	10	212	3.07E−04

**Figure 4:**
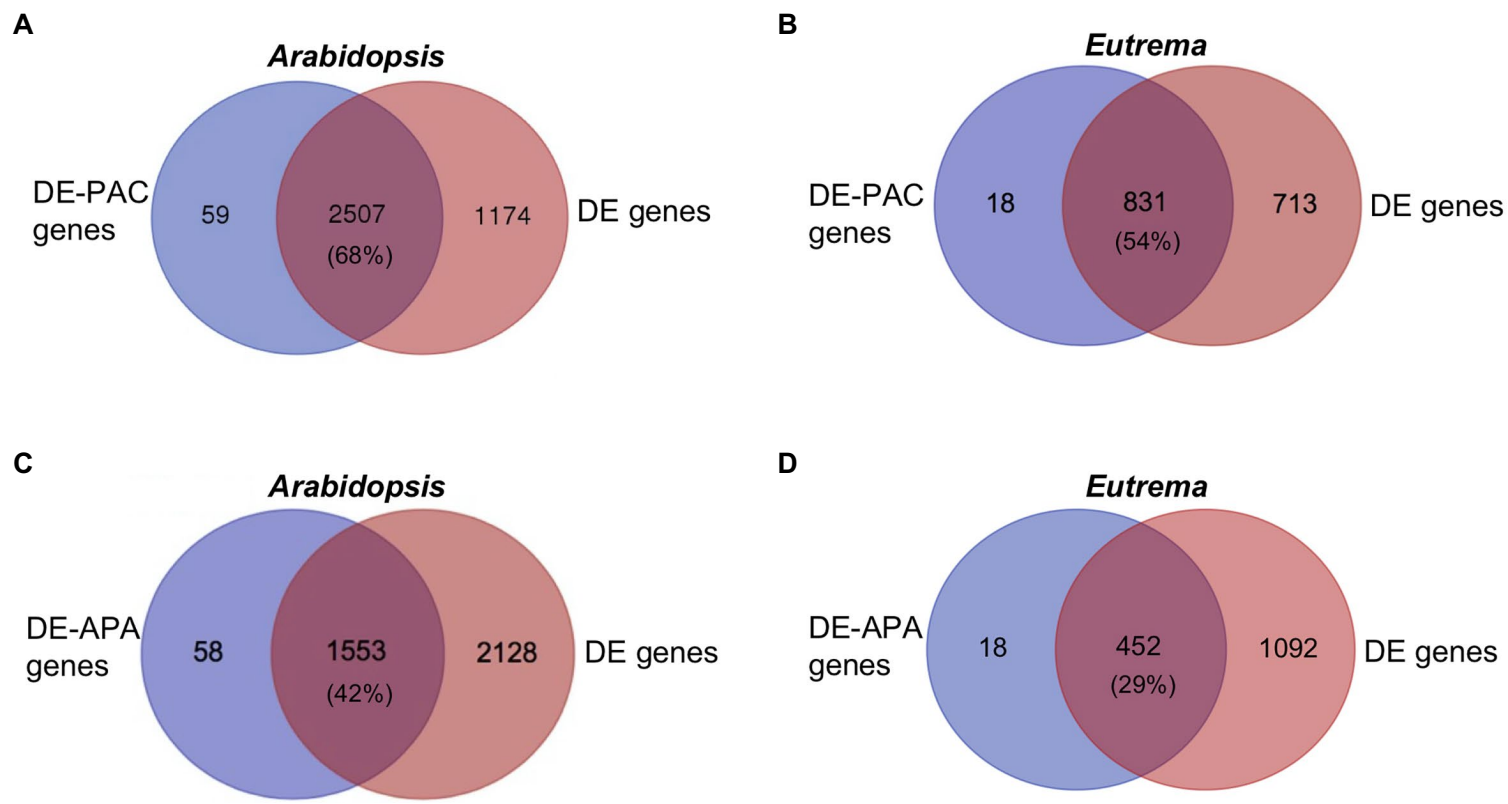
Venn diagrams for DE-PAC genes, differentially expressed APA genes (DE-APA genes), and differentially expressed genes (DE genes). **(A,B)** The overlap of DE-PAC genes and DE genes under salt stress in *Arabidopsis* and *Eutrema*. Numbers indicate gene numbers. Percentages indicate the fraction of DE genes overlapping with DE-PAC genes. **(C,D)** The overlap of DE-APA genes and DE genes under salt stress in *Arabidopsis* and *Eutrema*. Percentages indicate the fraction of DE genes overlapping with DE-APA genes.

Gene expression levels were determined by adding total counts of PATs located in the gene. Compared to CK, 3,681 genes in *Arabidopsis* and 1,544 genes in *Eutrema* were differentially expressed (DE) under ST. Venn analysis showed that 68% and 54% of the DE genes in *Arabidopsis* and *Eutrema*, respectively, had DE-PACs (Figures 4A,B). DE-PAC genes with more than one poly(A) site were defined as DE-APA genes. We found that a significant proportion of DE genes overlapped with DE-APA genes [42% in *Arabidopsis* (Figure 4C) and 29% in *Eutrema* (Figure 4D)], thus highlighting the importance of APA in the regulation of gene expression in response to salt stress.

### Genes Tended to Use Distal Poly(A) Sites in 3′ UTRs Under Salt Stress

**Figure 5:**
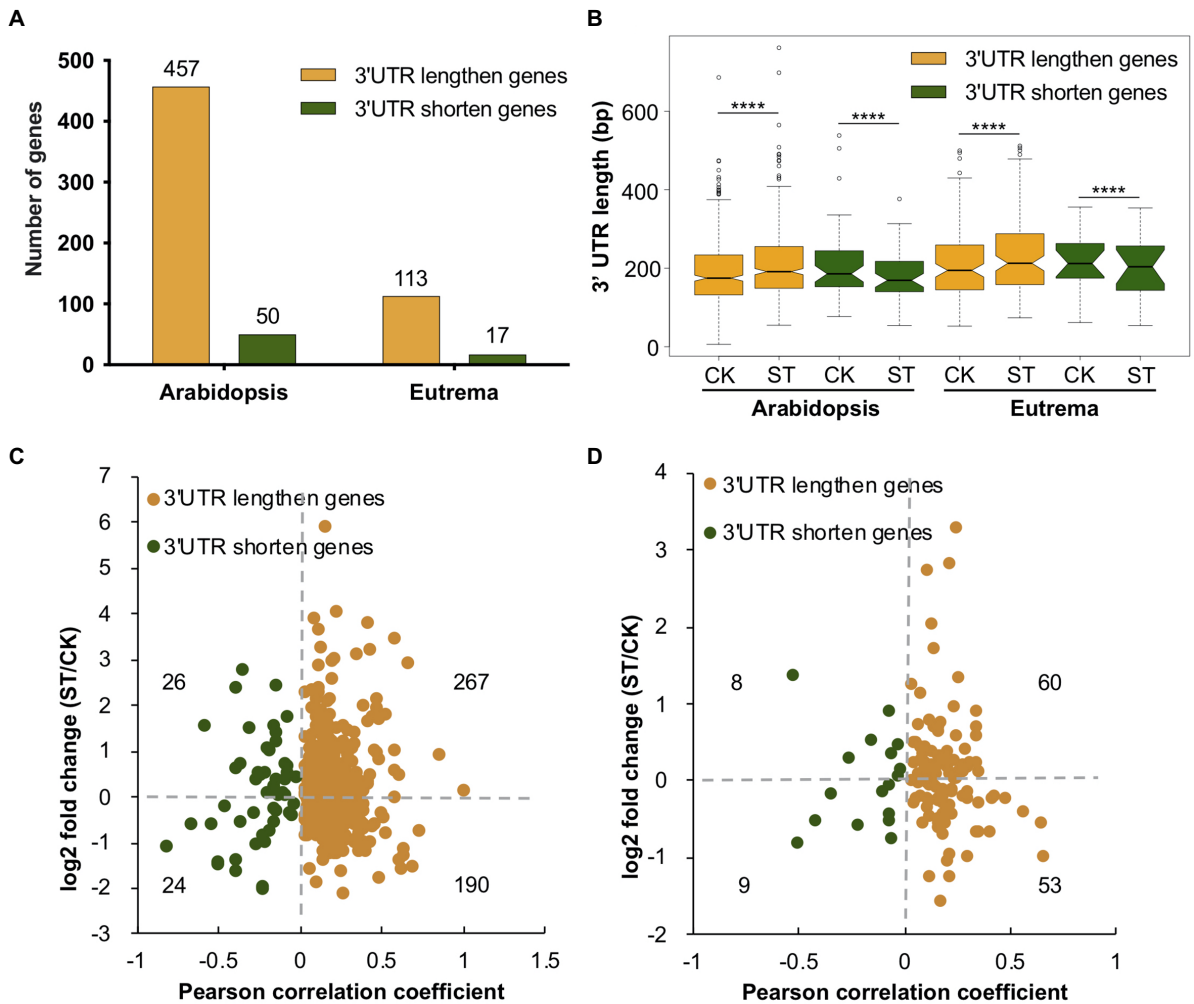
3′ UTR length analysis in *Arabidopsis* and *Eutrema*. **(A)** The number of genes showing changes in the length of 3′ UTR under salt stress. **(B)** 3′ UTR length of 3′ UTR changing genes under control (CK) and salt stress (ST) conditions. Statistical significance was determined by the Wilcoxon matched-pairs signed rank test, ^****^p < 10e−04. **(C,D)** Relationships between 3′ UTR length and gene expression level in *Arabidopsis* and *Eutrema*, respectively. Numbers indicate the number of genes. The *X*-axis indicates the strength of the change in the length of 3′ UTR; Pearson correlation coefficient > 0 indicates a longer 3′ UTR, Pearson correlation coefficient < 0 indicates a shorter 3′ UTR. The *Y*-axis indicates gene expression level; log2 fold change > 0 indicates upregulation, log2 fold change < 0 indicates down-regulation.

The 3′ UTR contains *cis*-elements that may affect mRNA metabolism, thus leading to the fine-tuning of mRNA stability, translation, nuclear export, and cellular localization (Xing and Li, 2011). Over 70% of PACs were located in 3′ UTRs of *Arabidopsis* and *Eutrema* (Figure 2D); therefore, we investigated APA events in this region and determined the length of 3′ UTRs in genes. These analyses suggested that there were a higher number of genes with longer 3′ UTRs than those with shorter 3′ UTRs in both *Arabidopsis* and *Eutrema* under salt stress. Compared to *Arabidopsis*, *Eutrema* possessed fewer genes that exhibited a change in the length of 3′ UTR (Figure 5A), thus indicating that 3′ UTR poly(A) sites were less affected in *Eutrema* under conditions of salt stress. Furthermore, we measured the 3′ UTR length of 3′ UTR lengthen and shorten genes in *Arabidopsis* and *Eutrema*. We found that salt stress caused significant changes in the length of 3′ UTR in both species (Figure 5B). Of the genes with longer 3′ UTRs, we found that more of these genes are upregulated than downregulated in *Arabidopsis* (267 vs. 190, with *p*_adj_ < 0.05, Figure 5C) and *Eutrema* (60 vs. 53, with *p*_adj_ < 0.05, Figure 5D). Of the genes with a shorter 3′ UTR, the numbers of upregulated genes and downregulated genes were very similar in both *Arabidopsis* (26 vs. 24) and *Eutrema* (8 vs. 9). The analysis of homologous genes with significantly longer 3′ UTRs in the two species showed that only eight genes overlapped (Supplementary Figure S3), thus revealing their distinct gene sets that responded to salt stress *via* APA in 3′ UTRs.

Next, we used GO analysis to investigate the functionality of genes undergoing significant changes in the length of their 3′ UTRs. No terms were enriched for the genes that exhibited shorter 3′ UTRs; this was most likely due to the limited number of genes; data related to the genes with longer 3′ UTRs are shown in Supplementary Figure S4. We found that the genes with a longer 3′ UTR in *Arabidopsis* were significantly enriched in GO terms related to salt stress, including response to salt stress and cation transport; such enrichment was not detected in *Eutrema*. These findings suggest that the regulation of APA in response to salt stress was more significant in *Arabidopsis* in terms of the poly(A) site choice in 3′ UTRs.

### Differential APA of Genes Related to Salt Tolerance in *Arabidopsis* and *Eutrema*

**Figure 6:**
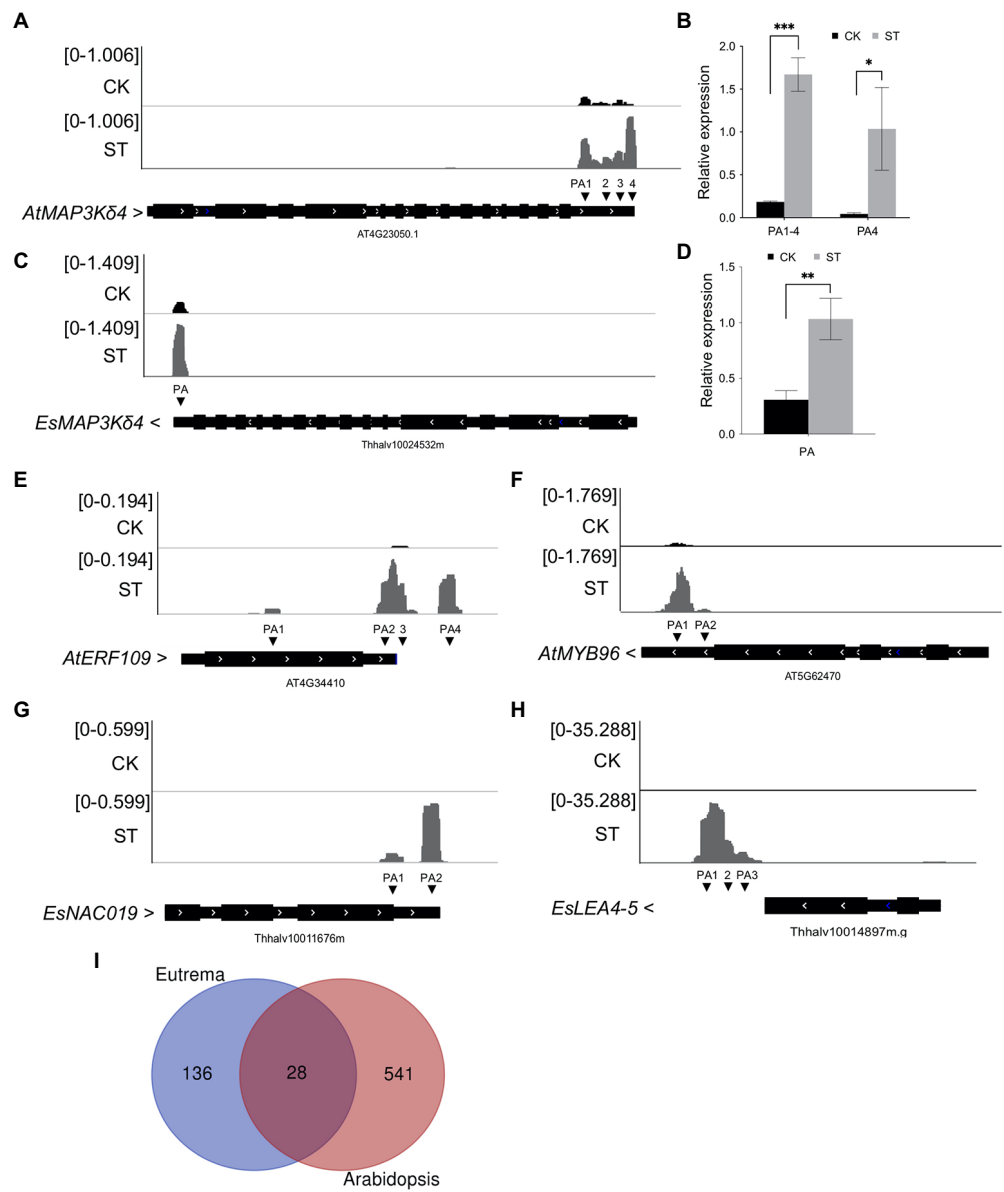
Differential APA in genes related to salt tolerance in *Arabidopsis* and *Eutrema*. **(A)** Integrative Genomics Viewer (IGV) showing the poly(A) sites of *AtMAP3Kδ4* (*AT4G23050*). CK, control; ST, salt stress. PA represents poly(A) site. Arrows beside gene names indicate gene orientation. **(B)** RT-qPCR was used to determine the expressive levels of distal poly(A) sites in *AtMAP3Kδ4*. **(C)** IGV showing the poly(A) site of *EsMAP3Kδ4* (*Thhalv10024532m*). **(D)** RT-qPCR was used to determine the expressive level of *EsMAP3Kδ4*. **(E)** IGV showing the poly(A) sites of *AtERF109* (*AT4G34410*). **(F)** IGV showing the poly(A) sites of *AtMYB96* (*AT5G62470*). **(G)** IGV showing the poly(A) sites of *EsNAC019* (*Thhalv10011676m*). **(H)** IGV showing the poly(A) sites of *EsLEA4-5* (*Thhalv10014897m*). **(I)** Venn plot showing the overlap of salt-specific PAC genes in *Arabidopsis* and *Eutrema*. Numbers indicate gene numbers. Statistical significance was determined by one-way ANOVA, ^*^p < 0.05, ^**^p < 0.01, and ^***^p < 0.001.

Interestingly, we found that some genes related to salt tolerance exhibited differential APA patterns in *Arabidopsis* and *Eutrema*. For example, MAP3Kδ4 plays an important role in ABA signaling and plant responses to various environmental stimuli, including high salt concentrations. The over-expression of *MAP3Kδ4* was previously shown to enhance tolerance to salt stress in *Arabidopsis* (Shitamichi et al., 2013). Our data further revealed that *AtMAP3Kδ4* (*AT4G23050*) exhibited a longer 3′ UTR under salt stress (from 280 nt in CK to 373 nt in ST). PAT-seq coverage of the gene was visualized by IGV and validated by RT-qPCR (Figures 6A,B). Four poly(A) sites were expressed under control conditions and the gene mostly used the proximal site (PA1). However, salt stress significantly increased the utilization of the distal poly(A) site (PA4, Figure 6A). The homolog of *AtMAP3Kδ4* in *Eutrema* (*Thhalv10024532m*) only showed increased gene expression level without APA regulation (Figures 6C,D).

When a gene exhibited alternative usage of two or more poly(A) sites (e.g., one PAC was upregulated while another was downregulated), then the gene was designated an APA switching gene. This type of APA switching under salt stress was detected in 70 and 23 genes in *Arabidopsis* and *Eutrema*, respectively. Table 2 shows APA switching genes for which a functional role has been described previously. In *Arabidopsis*, these genes are related to response to salt stress, mRNA processing, and growth by gravitropism. In *Eutrema*, these genes are related to dehydration stress, low temperature, and ABA response. It was previously reported that *ERD14* and *ERD10* were alternatively spliced following salt treatment (Ding et al., 2014) and that *erd10* mutants exhibited a reduced tolerance to dehydration (Kim and Nam, 2010). The homologous gene of *Thhalv10008280m* in *Arabidopsis* encodes AtU2AF35a, a small subunit of splicing factor U2. Interestingly, the gene that encodes the conserved subunit AtU2AF35b (*AT5G42820*) also underwent APA switching under salt stress in *Arabidopsis* (Table 2).

**Table 2:** APA switching genes and their functions from previous studies.

*Arabidopsis*			
Gene	Name	Function	References
*AT4G29820*	*CFIM-25*	Encodes a homolog of the protein CFI-25, a polyadenylation factor subunit.	Hunt et al., 2008
*AT1G24706*	*THO2*	Small RNA biosynthesis.	Francisco-Mangilet et al., 2015
*AT5G42820*	*U2AF35B*	U2 auxiliary factor small subunit.	Wang and Brendel, 2006
*AT5G57630*	*CIPK21*	Response to salt stress.	Pandey et al., 2015
*AT1G70940*	*PIN3*	A regulator of auxin efflux and involved in differential growth; gravitropism.	Rakusová et al., 2016
***Eutrema***			
Gene	Name of Arabidopsis homologs	Function	References
*Thhalv10019152m*	*ERD14*	Induced early on in response to dehydration stress	Kiyosue et al., 1994
*Thhalv10008313m*	*ERD10*	Induced by low temperature and dehydration	Kim and Nam, 2010
*Thhalv10008280m*	*U2AF35A*	U2 auxiliary factor small subunit	Wang and Brendel, 2006
*Thhalv10001457m*	*CIR*	Pre-mRNA splicing factor	Maita et al., 2005
*Thhalv10003590m*	*NPX1*	A nuclear factor regulating abscisic acid responses	Kim et al., 2009

In addition, considering stress conditions can induce the specific expression of genes, we investigated salt-specific PACs (i.e., PACs that were only expressed in ST samples) and salt-inducible APA (i.e., APA events that were only found in ST samples) in *Arabidopsis* and *Eutrema*. In total, 1,021 salt-specific PACs were identified in *Arabidopsis*, these were dispersed among 569 genes; 86 of these genes were enriched in GO terms related to transcription factors and 46 genes were enriched in GO terms related to response to salt stress. Notably, 50 genes showed salt-inducible APA; furthermore, some transcription factors that positively regulate drought and salt stress only underwent APA under conditions of salt stress. For example, *AT4G34410* only used one poly(A) site under normal conditions, whereas four PACs were induced by salt stress (Figure 6E). This indicated that salt stress changed the poly(A) tailing position of *AT4G34410* transcripts. This gene encodes the transcription factor ERF109, which improves the resistance of *Arabidopsis* to salt. Compared with knockout mutants, mutants that overexpressed ERF109 were shown to possess a longer root length, more leaves, and larger rosette leaf areas under salt conditions (Bahieldin et al., 2016). Another gene, *AT5G62470* is known to encode the MYB96 transcription factor; in this gene, only one poly(A) site was used in the absence of salt stress, while two PACs were produced under salt stress (Figure 6F). MYB96 transcription factor has been shown to improve tolerance to drought in *Arabidopsis* by regulating the biosynthesis of cuticular wax (Seo et al., 2011).

In *Eutrema*, we identified 190 salt-specific PACs from 169 genes. Of these genes, 18 were significantly enriched in GO terms related to transcription factor activity and sequence-specific DNA binding; 14 were enriched in response to water deprivation. Sixteen genes showed salt-inducible APA; likewise, some transcription factors that positively regulate drought and salt stress only exhibited APA under salt stress. These included *Thhalv10011676m*, which encodes a homolog of *Arabidopsis* NAC019 transcription factor; this gene did not undergo expression under normal conditions but produced two PACs following salt treatment (Figure 6G). *Thhalv10014897m* encodes a homolog of *AtLEA4-5* that typically accumulates in response to conditions of low water availability (Li et al., 2021b). This gene exhibited only one PAC in the absence of salt but exhibits three PACs under salt stress (Figure 6H). Moreover, we used salt-specific PAC genes in *Eutrema* to identify homologous genes in *Arabidopsis* for comparative purposes. Venn analysis showed that only 28 genes overlapped (Figure 6I); these genes were significantly enriched in GO terms related to water deprivation, response to abscisic acid, and transcription factor activity. However, more salt-specific PAC genes in *Arabidopsis* are distinct from that in *Eutrema*, thus suggesting that APA plays an important role in both species during salt stress response but with different patterns of gene regulation; a higher number of salt-specific PACs were activated in *Arabidopsis* to cope with salt conditions.

### Polyadenylation Factors Exhibited Different Expression Levels Under Salt Stress

**Figure 7:**
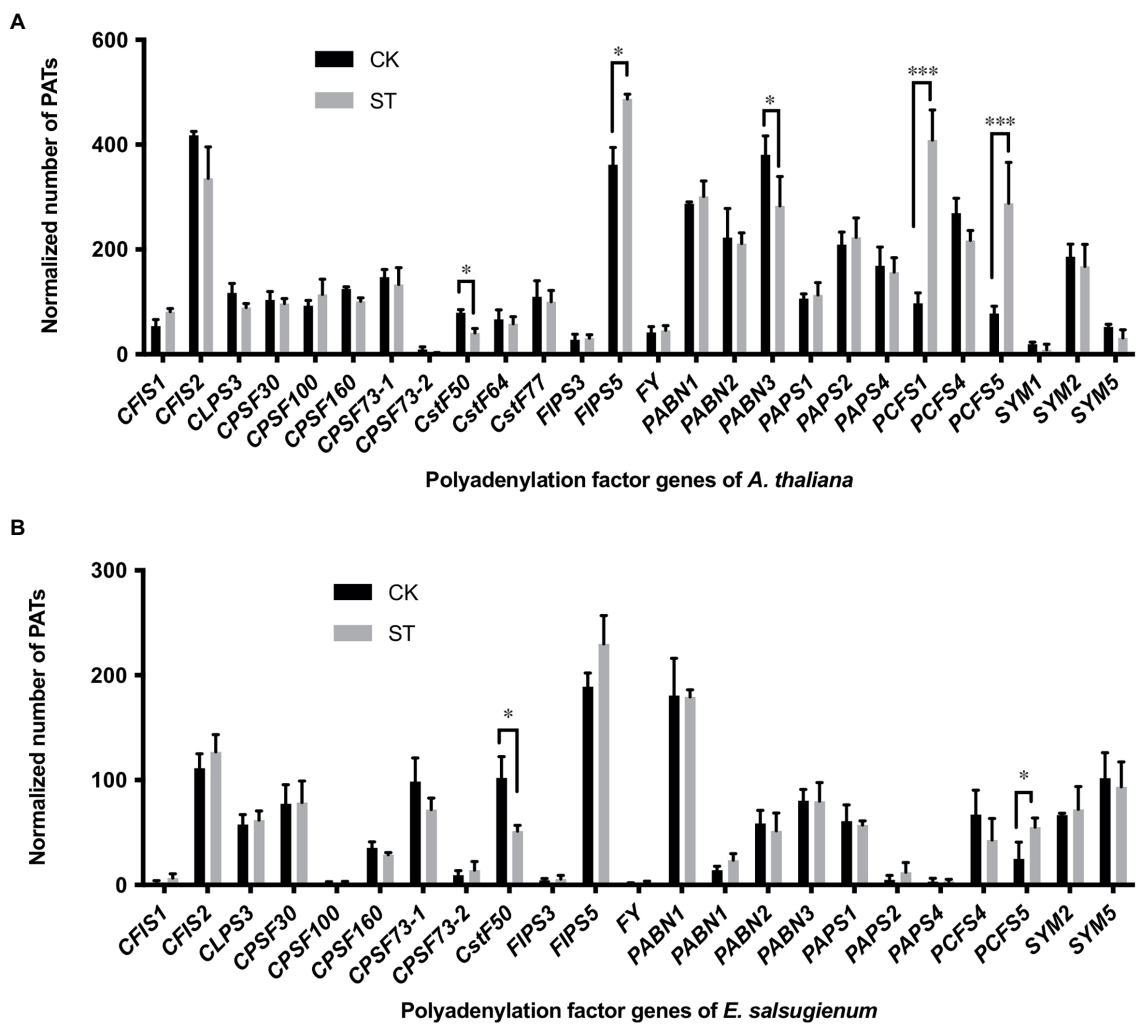
Gene expression levels of polyadenylation factors in *Arabidopsis* **(A)** and *Eutrema* **(B)**. CK, control; ST, salt stress. Statistical significance was determined by one-way ANOVA, ^*^p < 0.05, ^***^p < 0.001.

The differential use of APA sites is normally related to the different functions of poly(A) factors. Changes in the expression of core polyadenylation factors will also lead to global APA events in 3′ UTRs (Thomas et al., 2012). To explore the mechanisms responsible for the modulation of 3′ UTR length, we determined the expression levels of 26 genes that encode polyadenylation factors and compared these data between CK and ST samples. In *Arabidopsis*, three polyadenylation factor genes (*FIPS5*, *PCFS1*, and *PCFS5*) were significantly upregulated under salt stress (Figure 7A). The homologous genes of *PCFS5* in *Eutrema* also showed upregulation under salt stress (Figure 7B); these data were consistent with previous studies that reported *AtPCFS1* and *AtPCFS5* to exhibit increased expression levels under salt stress (Hunt et al., 2008). In contrast, *CstF50* and *PABN3* were significantly downregulated in *Arabidopsis* under salt stress (Figure 7A); while *CstF50* was downregulated in *Eutrema* (Figure 7B). PCFS factors are homologs of Pcf11p in yeast and *CF* II in mammals and are essential for pre-mRNA 3′-end processing. Yeast Pcf11p binds to the C-terminal domain of the largest subunit of RNA polymerase II and is involved in transcription termination, and its C-terminal part interacts with polyadenylation factor Clp1p, Rna14p, and Rna15p (Haddad et al., 2012). In mammals, CstF50 is a subunit of the cleavage stimulation factor complex and interacts with BRCA1-associated RING domain protein to inhibit polyadenylation *in vitro* (Kleiman and Manley, 1999). In *Arabidopsis*, CstF50 interacts with CstF64, PAPS, and CPSF factors (Hunt et al., 2008). Therefore, polyadenylation factors may play important roles in salt-induced APA by interacting with other polyadenylation factors and by modulating the expression of genes that are responsive to salt stress.

**Figure 8:**
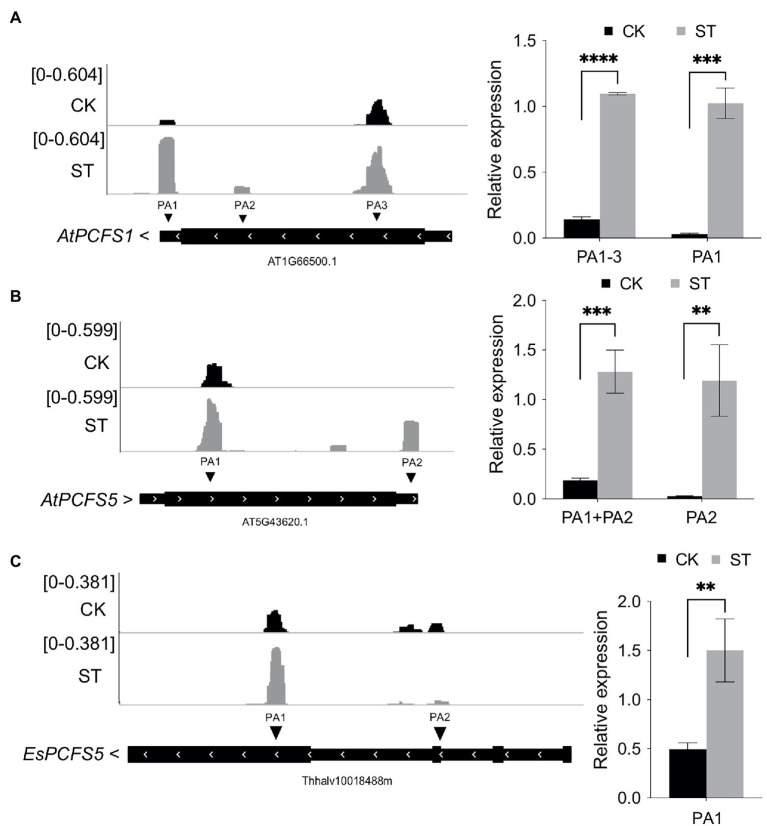
The poly(A) tag sequencing (PAT-seq) coverage of PCFS factor genes by IGV and RT-qPCR results. **(A)** The PAT-seq coverage of *AtPCFS1* (*AT1G66500*) and RT-qPCR result. **(B)** The PAT-seq coverage of *AtPCFS5* (*AT5G43620*) and RT-qPCR result. **(C)** The PAT-seq coverage of *EsPCFS5* (*Thhalv10018488m*) and RT-qPCR result. CK, control; ST, salt stress. PA represents poly(A) site. Arrows beside gene names indicate gene orientation. Statistical significance was determined by one-way ANOVA, ^**^p < 0.01, ^***^p < 0.001, and ^****^p < 10e−04.

The abundance of transcripts of *PCFS* factor genes were visualized by IGV and the gene expression levels were validated by RT-qPCR. Under control conditions, *AtPCFS1* (*AT1G66500*) mainly used the poly(A) site located in the CDS region; however, under conditions of salt stress, the use of the distal poly(A) site in the 3′ UTR increased dramatically (Figure 8A). A similar phenomenon was also evident for *AtPCFS5* (*AT5G43620*, Figure 8B). Interestingly, the homologous gene of *AtPCFS1* and *AtPCFS5* in *Eutrema*, *EsPCFS5* (*Thhalv10018488m*), also showed increased expression level of the distal poly(A) site under salt stress (Figure 8C). These results suggest that *Arabidopsis* and *Eutrema* might use APA to increase the expression levels of functional transcripts of polyadenylation factors in response to salt stress.

## Discussion

In this study, we provide a comprehensive map of poly(A) profiles of a salt-sensitive species (*A. thaliana*) and a salt-tolerant species (*E. salsugineum*), and compare their APA patterns under salt stress. Although APA occurs commonly in *Arabidopsis* and *Eutrema*, *Arabidopsis* possesses a higher number of APA genes than *Eutrema* (54% vs. 47%). Furthermore, the proportion of APA genes increased significantly in *Arabidopsis* under salt stress, but not in *Eutrema*. Both species tend to use distal poly(A) sites under salt stress, while their 3′ UTR lengthen genes showed different enrichments in GO terms and KEGG pathways. Salt stress affected the use of poly(A) sites within 3′ UTRs in a larger number of genes in *Arabidopsis* than in *Eutrema* (507 vs. 130). *Eutrema* exhibits an innate response to salt stress; therefore, gene expression was less affected in this species. APA was found to be associated with 42% and 29% of DE genes in *Arabidopsis* and *Eutrema* under salt stress, respectively, thus suggesting the potential role of APA in the regulation of gene expression in response to salt stress. Salt-specific PACs and salt-inducible APA events were identified in both species; interestingly, some genes related to salt tolerance and transcription factor genes showed differential APA patterns. Our results suggest that the more adaptive species showed less alteration at the transcriptional level under stress while more salt-specific PACs were activated in *Arabidopsis* to cope with salt conditions.

### Polyadenylation Factors and Wide-Ranging APA Under Stress Conditions

A large group of protein factors are required for pre-mRNA polyadenylation process in plants. These factors recognize polyadenylation signals and form complexes that control mRNA 3′-end formation. The polyadenylation factor subunits not only show extensive protein–protein interactions, but also coordinate with other RNA processing events in the course of gene expression (Hunt et al., 2008). Previous studies on AtCPSF30, AtCPSF100 and FY suggested that changes in the activity of polyadenylation factors may lead to wide-ranging APA (Thomas et al., 2012; Lin et al., 2017; Yu et al., 2019). In addition, abiotic stress treatments can incite changes in poly(A) site choice in a large number of genes. Some APA patterns have been shown to change extensively under abiotic stresses, including drought, heat, and salt stress in *Sorghum* (Chakrabarti et al., 2020); oxidative stress (Liu et al., 2014), and hypoxia in *Arabidopsis* (de Lorenzo et al., 2017); drought, heat shock, and cadmium stress in rice (Ye et al., 2019). Interestingly, abiotic stresses tend to increase the usage of non-canonical poly(A) sites in plants (de Lorenzo et al., 2017; Chakrabarti et al., 2020). In our study, by comparing the expression levels of polyadenylation factors under control and salt stress conditions in *Arabidopsis* and *Eutrema*, we found that five polyadenylation factors in *Arabidopsis* changed significantly in their expression levels when responded to salt stress, whereas only two polyadenylation factors in *Eutrema* showed significant changes (Figure 7). Notably, AtPCFS1 and AtPCFS5 showed highly significant changes. Moreover, the expression levels of many polyadenylation factors of *Eutrema* were lower than that of *Arabidopsis* in both stressed and unstressed conditions. Thus, the changes in the expression levels of core polyadenylation factors in *Arabidopsis* may widely affect the selection and usage of poly(A) sites during the salt stress response. Meanwhile, most polyadenylation-related genes in *Eutrema* responded modestly. This may explain the result that more APA events were identified in *Arabidopsis* in response to salt stress than that in *Eutrema*.

### Consequences of APA in Different Regions of Genes Under Stress

Alternative polyadenylation that happened in different regions of genes would lead to various stabilities of mRNAs. Those mRNAs generated by polyadenylation in CDS regions, which lack stop codons, are likely to be degraded through non-stop mRNA decay pathways; and the mRNAs end in introns may be targeted by nonsense-mediated decay pathway (Frischmeyer et al., 2002). Interestingly, the process of mRNA degradation could be downregulated under stress conditions (Shaul, 2015), thereby promoting the accumulation of non-canonical mRNAs. This brings a possible explanation to the increase in non-canonical isoforms in response to stresses. Within the APA events in 3′ UTRs, we identified more genes possess longer 3′ UTRs rather than shorter 3′ UTRs, and a larger proportion of the 3′ UTR lengthen genes showed significantly upregulation under salt stress. This is consistent with the findings reported previously. UV light caused DNA damage in the *Saccharomyces cerevisiae* gene and led to changes in poly(A) sites along with the extension of transcripts (Graber et al., 2013). Another study reported that osmotic stress caused by KCl in human fibroma cells, along with dehydration stress in *Arabidopsis*; both resulted in 3′ UTR extension to nuclear chromatin combination areas and long non-coding regions (Vilborg et al., 2015; Proudfoot, 2016). These findings indicate that repression of the proximal poly(A) sites and utilization of the distal poly(A) sites in 3′ UTRs might be a general mechanism for stress responses. Although 3′ UTR-APA does not change the coding sequence or total expression levels of mRNA, this process may affect post-transcriptional gene regulation in various ways, including mRNA stability, the modulation of mRNA translation, nuclear export, cellular localization, and the localization of encoded proteins (Berkovits and Mayr, 2015; Tian and Manley, 2017). We did observe gene expression level changes in some translation elongation factors such as *TFIIS*; this may have had an impact on mRNA translation efficiency by altering the use of polyadenylated transcripts (Cui and Denis, 2003).

### APA As a Part of Response to Stresses or Disorder?

In the current study, the poly(A) profiles of *Arabidopsis* were widely affected by abiotic stress. This phenomenon also exists in several prior observations of different plant species upon exposure to abiotic stresses (Zhang et al., 2008; de Lorenzo et al., 2017; Ye et al., 2019; Chakrabarti et al., 2020). Whether APA is a part of the regulatory network in response to stresses or it is a disorder of RNA processing induced by stresses becomes an interesting question. Firstly, there are indeed examples to show that APA plays a role in plant stress responses. It was demonstrated *in vivo* that the transcripts of *AtARK2* and a transcriptional regulator gene generated by APA play roles in salt stress and oxidative stress responses (Yu et al., 2019). Secondly, multiple polyadenylation factors have been reported to be associated with abiotic or biotic stress responses, including CPSF30, FIP1, FY, and CPSF100 (Liu et al., 2014; Lin et al., 2017; Tellez-Robledo et al., 2019; Yu et al., 2019), suggesting the potential role of APA mediated by polyadenylation factors. Besides, many APA switching events we identified in this study and previous studies are related to stress response genes. For example, many APA switching genes were found in rice samples of different tissues and developmental stages, and these genes have functions related to salt and drought stress responses (Fu et al., 2016). This indicates that APA not only regulates the developmental process of plants, but also regulates the adaptation process of plants to abiotic stresses. Furthermore, it is well studied that stresses incite the expression of many stress-related genes (Chen et al., 2015). Similarly, we herein observed a large group of stress-responsive poly(A) sites and APA events. That said APA under salt stress could provide extensive plasticity for the plants to adapt to stress conditions.

On the other hand, stresses may reduce the fraction of 3′ UTR poly(A) sites and lead to an increase of non-canonical poly(A) sites, as we observed in *Arabidopsis* when exposed to salt conditions. This is consistent with prior observations showing that the usage of poly(A) sites in CDS, intron and 5′ UTR regions were promoted by salt, drought, heat treatment, and hypoxia (de Lorenzo et al., 2017; Tellez-Robledo et al., 2019; Chakrabarti et al., 2020). Notably, the isoforms end in CDSs and introns were less stable and underrepresented in polysomes; conversely, transcripts generated by 5′ UTR poly(A) sites were as stable as canonical isoforms (de Lorenzo et al., 2017). Nevertheless, the re-directing of transcriptional output may represent a form of negative regulation under stresses. Some researchers believed that the stress-inducible remodeling of transcripts mediated by APA represents an important part of the regulatory network in plant stress responses (Chakrabarti et al., 2020).

Collectively, we believe that APA plays a functional role in the regulatory response to stresses. Although the contribution of genome-wide changes mediated by APA requires further exploration, they may need to be considered carefully on a case-by-case basis.

## Conclusion

*Eutrema* has adapted to salty environments throughout its evolutionary history while *Arabidopsis* has not. In the present study, comparison of their poly(A) site usage (reflecting RNA processing) under salt stress revealed that their responses are distinct in that *Eutrema* are relatively stable while *Arabidopsis* shows significant changes in gene expression *via* APA. These results are suggestive that innate responses to environmental insults in plants relate to inherited ability. Such ability could be written into the genetic circuits for gene expression in a particular species. Further elucidation of these circuits would be of significant benefit to the genetic engineering of crops.

## Data Availability Statement

The datasets presented in this study can be found in online repositories. The names of the repository/repositories and accession number can be found at: https://www.ncbi.nlm.nih.gov/, PRJNA782687.

## Author Contributions

LC and KZ prepared the plant materials and salt treatments, and LC made PAT-seq libraries. HM and LC performed the data analyses and prepared the manuscript. JL participated in the data analyses and revised the manuscript. QL conceived and supervised the project and revised the manuscript. All authors contributed to the article and approved the submitted version.

## Funding

This research was supported in part by a grant from Chinese Ministry of Science and Technology (2016YFE0108800). HM received funding support from China Scholarship Council while visiting Western University of Health Sciences.

## Conflict of Interest

The authors declare that the research was conducted in the absence of any commercial or financial relationships that could be construed as a potential conflict of interest.

## Publisher’s Note

All claims expressed in this article are solely those of the authors and do not necessarily represent those of their affiliated organizations, or those of the publisher, the editors and the reviewers. Any product that may be evaluated in this article, or claim that may be made by its manufacturer, is not guaranteed or endorsed by the publisher.

## Footnotes

^1^ https://www.ncbi.nlm.nih.gov/
