# Supplementary material for: Divergence in the Regulation of the Salt Tolerant Response Between Arabidopsis thaliana and Its Halophytic Relative Eutrema salsugineum by mRNA Alternative Polyadenylation
Source: Front Plant Sci. 2022 Mar 25;13:866054. doi: 10.3389/fpls.2022.866054 (PMC8993227; doi:10.3389/fpls.2022.866054)
Supplement: Supplementary file 1 [file Data_Sheet_1.pdf]

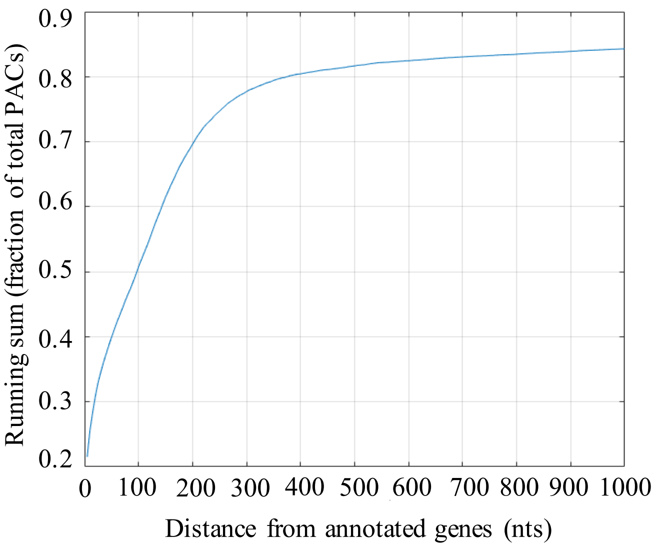

Figure S1. The fraction of PACs within 1,000 nts downstream of 3′ UTRs (intergenic regions) in *Eutrema*.

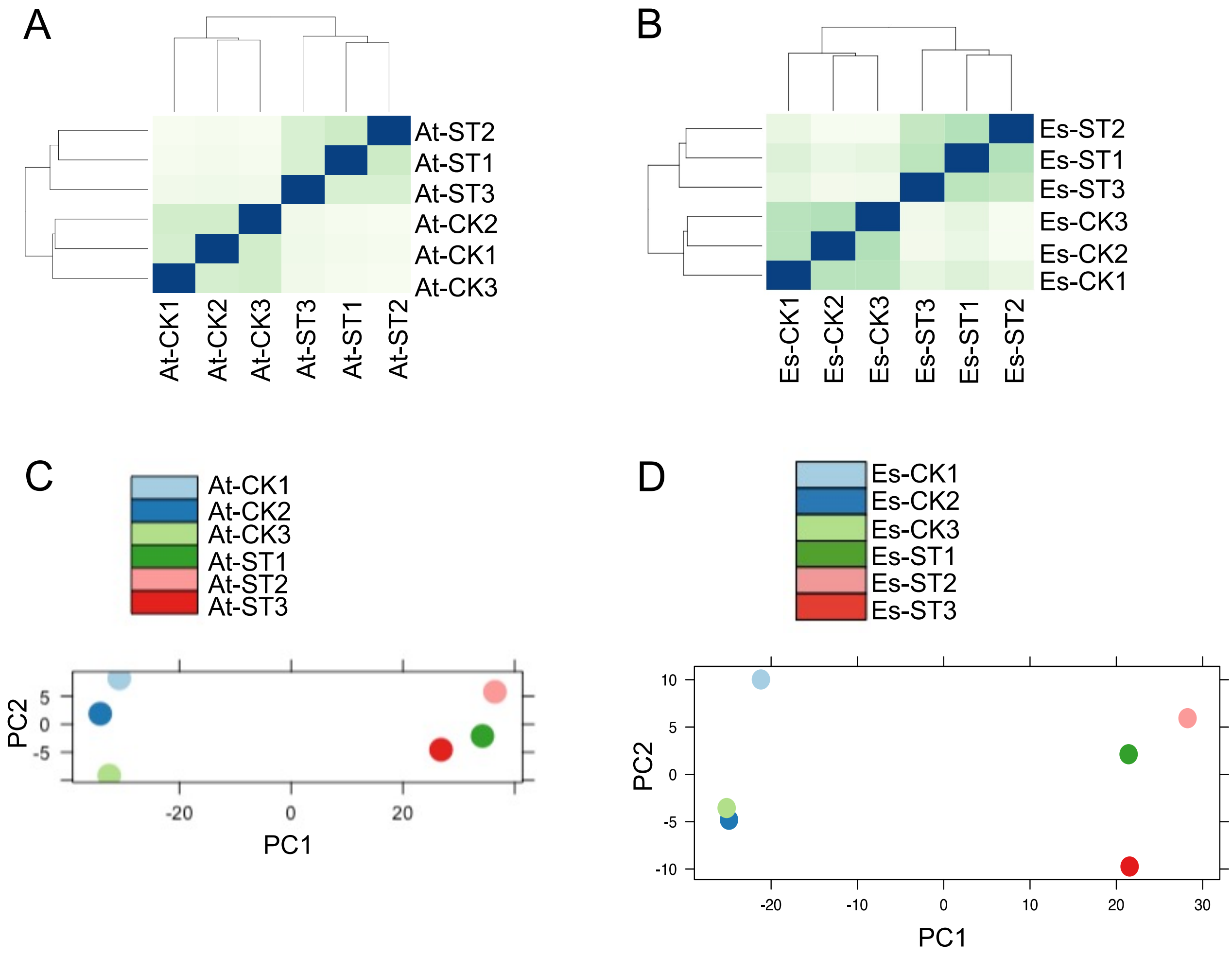

Figure S2. Sample distances and principal component analysis of *Arabidopsis* and *Eutrema* samples. (**A**) **and** (**B**) Heat maps showing the Euclidean distance between samples of *Arabidopsis* and *Eutrema*. (**C**) **and** (**D**) Principal component analysis showing PAT-seq samples of *Arabidopsis* and *Eutrema*. At, Arabidopsis; Es, Eutrema. CK, control; ST, salt stress.

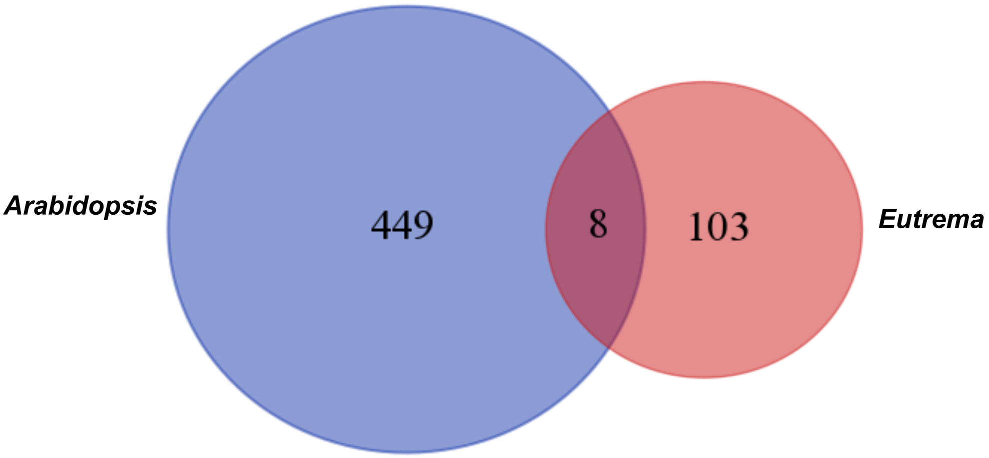

Figure S3. Homologous analysis of 3′ UTR lengthen genes in *Arabidopsis* and *Eutrema.* Numbers indicate gene numbers.

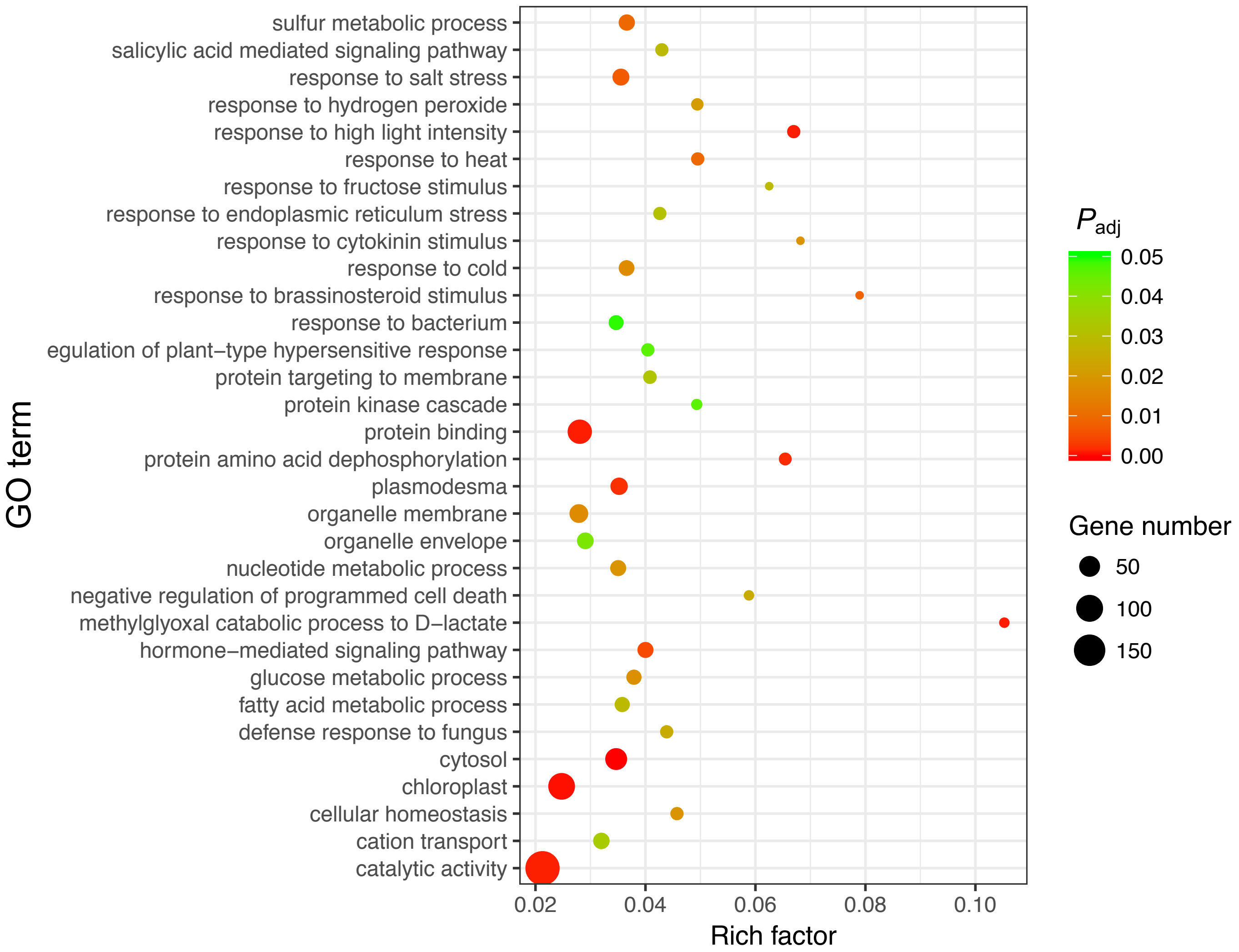

Figure S4. GO analysis of 3' UTR lengthen genes in *Arabidopsis.* Rich factor indicates the gene number annotated in the term divide by the gene number in reference annotation. P_{adj} , adjusted P value.

Table S3. Primer list.

Primer	Sequence (5’-3’)
*AtPCFS1*_PA3_F	TCAGATCACAAGCGCAACAG
*AtPCFS1*_PA3_R	AGCAATCACATCAACACCGC
*AtPCFS1*_PA1_F	CGTAGAGCCGTTTGAAGAGTT
*AtPCFS1*_PA1_R	ATCCTTAGCAGGTCGGGGTT
*AtPCFS5*_PA1_F	AACGGATCCTTCTCTGCTCAG
*AtPCFS5*_PA1_R	TAACATCCGATTCCGCCACC
*AtPCFS5*_PA2_F	AAGTGTATGCCTGAACCCCG
*AtPCFS5*_PA2_R	ATTACCCTCGACGGCTCTCT
*EsPCFS5*_PA_F	GGAGTTTTCCAGGAGGATCG
*EsPCFS5*_PA_R	TTGGAATCTGGTGCGTTTC
*AtMAP3Kδ4*_PA1_F	AGAGTGGTTTGGTGTCTGGC
*AtMAP3Kδ4*_PA1_R	GGTTGACAGGACTTTGTTGCC
*AtMAP3Kδ4*_PA3_F	AGGAGTGACAATTCGTAGGCA
*AtMAP3Kδ4*_PA3_R	GAGAAGCTACAGACGACGCA
*EsMAP3Kδ4*_PA_F	GCGAGACTAAGGAACGATGTG
*EsMAP3Kδ4*_PA_R	GATGCCGATTCGTCTATCCT
